# Left ventricular function assessment after aortic and renal intervention in Takayasu arteritis by speckle tracking echocardiography: A pilot study

**DOI:** 10.1016/j.ihj.2022.02.004

**Published:** 2022-02-23

**Authors:** Sudhir Mor, Sanjay Tyagi, Shekhar Kunal, Ankit Bansal, M.P. Girish, Vishal Batra, Mohit Dayal Gupta

**Affiliations:** Department of Cardiology, Govind Ballabh Pant Institute of Post Graduate Medical Education and Research, Delhi, India

**Keywords:** Ejection fraction, Global longitudinal strain, Speckle tracking echocardiography, Subclinical left ventricle dysfunction, Takayasu arteritis

## Abstract

**Background:**

Overt left ventricular (LV) dysfunction and congestive heart failure are known entities in Takayasu arteritis (TA). Subclinical LV dysfunction may develop in these patients despite normal LV ejection fraction (LVEF). Moreover, effect of treatment of aortic or renal artery narrowing in such patients is unknown.

**Methods:**

This study included 15 angiographically confirmed TA patients undergoing aortic and/or renal intervention. A comprehensive clinical, biochemical and echocardiographic (2-dimensional, speckle tracking and tissue doppler imaging) evaluation were done at baseline, 72 h, and six months post intervention.

**Results:**

Six patients (40%) had reduced LVEF (<50%) at baseline while rest 9 (60%) patients had reduced global longitudinal strain (GLS) but normal EF. Diastolic filling pattern was abnormal in all the patients. In patients with baseline reduced EF, mean EF improved from 24.62 ± 12.14% to 45.6 ± 9.45% (*p* = 0.001), E/e’ ratio decreased from 15.15 ± 3.19 to 10.8 ± 2.56 (*p* = 0.005) and median NT pro BNP decreased from 1673 pg/ml (970–2401 pg/ml) to 80 pg/ml (40–354 pg/ml) (*p* = 0.001) at 6 months after interventional procedure. In patients with baseline normal EF, median NT pro BNP decreased from 512 pg/ml (80–898.5 pg/ml) to 34 pg/ml (29–70.8 pg/ml) (*p* < 0.01), mean GLS improved from −8.80 ± 0.77% to −16.3 ± 0.78% (*p* < 0.001) and mean E/e’ decreased from 12.93 ± 2.63 to 7.8 ± 2.73 (*p* = 0.005) at 6 months follow up.

**Conclusion:**

LV dysfunction is common in patients with TA and obstructive lesions in aorta or renal arteries. GLS can be used to assess subclinical systolic dysfunction in these patients. Timely intervention can improve LV dysfunction and can even reverse the subclinical changes.

## 1 Introduction

Takayasu arteritis (TA) is an idiopathic chronic inflammatory disease of the aorta and its major branches leading to stenosis, occlusion and aneurysmal dilatation.^1^ Congestive heart failure (CHF) is the major cause of death in patients with TA.^2^ The causes of left ventricular (LV) dysfunction in TA include renal artery stenosis, aortic obstruction, myocarditis, aortic regurgitation and coronary artery involvement.^3^, ^4^, ^5^ Post intervention amelioration of symptoms, reduction in hypertension and improvement in LV systolic function have been suggested in case reports and small case series.^6^, ^7^, ^8^, ^9^ Conventional echocardiography usually detects manifest LV dysfunction following a prolonged disease process, however, speckle tracking echocardiography (STE) being a more sensitive tool can detect sub-clinical myocardial dysfunction even in the presence of preserved systolic function. This pilot study assessed effect of aortic and renal intervention on systolic and diastolic function by conventional echocardiography as well as STE in patients with TA. To the best of our knowledge, there has been no published data documenting the utility of STE to determine the global longitudinal strain (GLS) in patients with Takayasu arteritis.

## 2 Methods

### 2.1 Study design

This was a single-center prospective observational study wherein consecutive patients with TA diagnosed as per the American College of Rheumatology (ACR) criteria^10^ and requiring aortic and/or renal intervention were enrolled. Patients of TA not undergoing aortic/renal interventions, vascular aneurysms, those with low-quality echocardiographic images, pre-existing cardiovascular diseases such as coronary artery disease, valvular heart disease, chronic obstructive pulmonary disease, prior cerebrovascular disease, chronic liver or kidney disease [(eGFR <30 ml/min/m^2^) and cardiac rhythm disturbances like atrial fibrillation or pacing were excluded. All patients underwent a detailed clinical assessment including symptomatology, determination of baseline clinical and biochemical parameters including estimated glomerular filtration rate (GFR), serum creatinine, inflammatory markers such as C-reactive protein (CRP) and erythrocyte sedimentation rate (ESR) and N-Terminal Pro brain Natriuretic Peptide (NT pro BNP). Additionally, CT aortogram as well as conventional angiography of the aorta and its branches were performed to determine the extent of the lesions. All these patients were on oral corticosteroids along with methotrexate once weekly pre and post intervention.

**Fig. 1:**
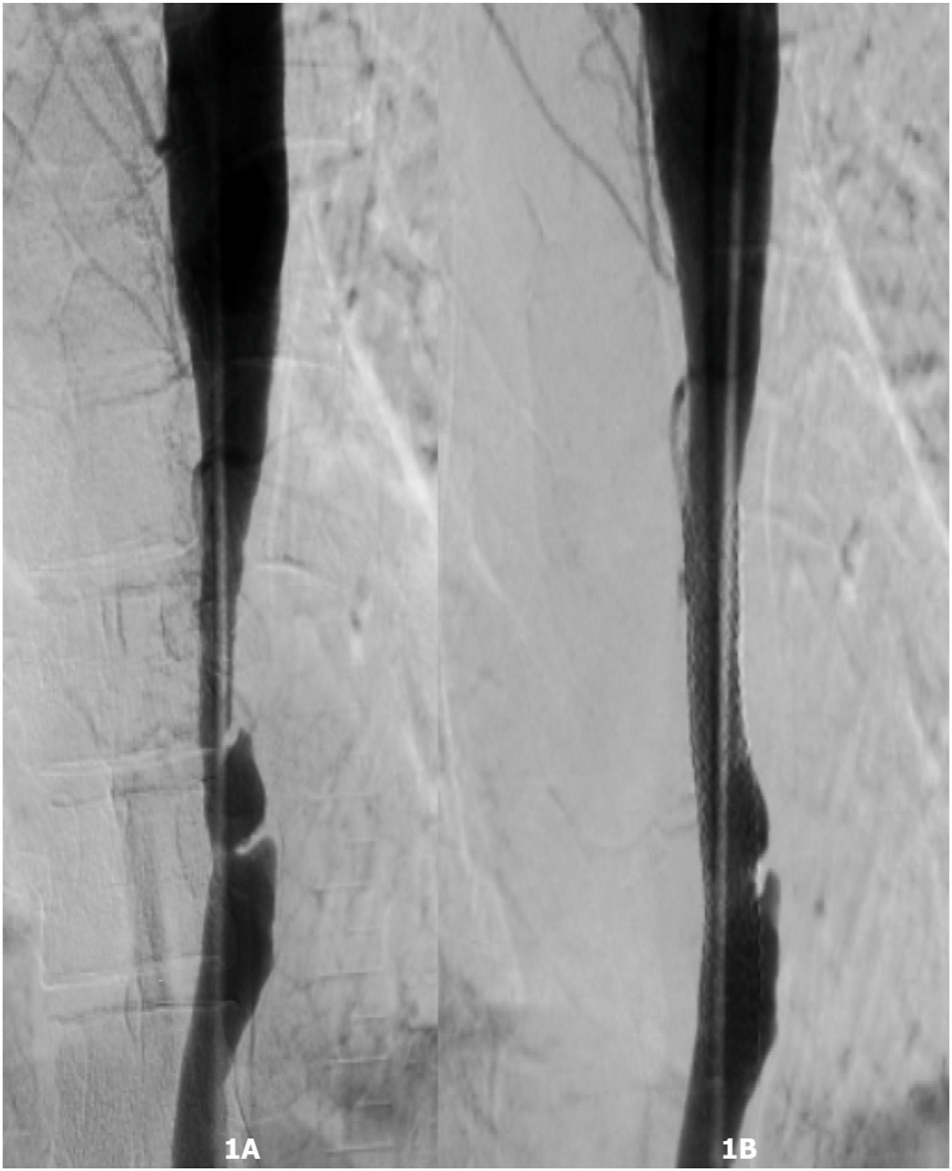
A Digital subtraction angiography (pre-intervention) from a 29 years old female showing the diffuse narrowing of the descending thoracic aorta. Fig. 1B: Digital subtraction angiography (post balloon dilation and stenting) showing relief of obstruction of the descending thoracic aorta with the mean gradient across the narrowing decreased from 100 mm Hg to 20 mm Hg post stenting.

Aortic intervention was done in symptomatic cases of aortic obstruction with a mean gradient greater than 20 mm Hg (Fig. 1) and renal intervention was done in cases of renal artery stenosis greater than 70% along with hypertension defined as blood pressure ≥130/80 mm Hg (ACC/AHA Hypertension guidelines, 2017).^11^

**Fig. 2:**
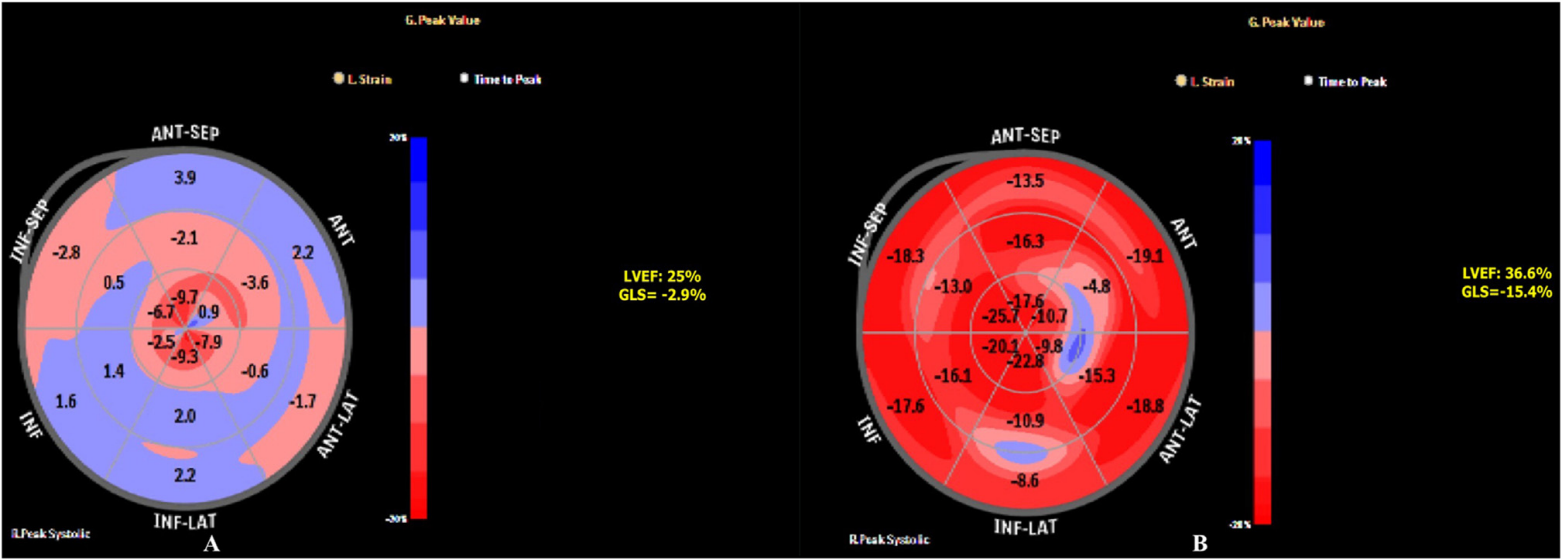
A Pre-intervention speckle tracking echocardiography bull's eye plot of the same patient as in Fig. 1A and B showing global longitudinal strain to be −2.9% and left ventricular ejection fraction of 25%. Fig. 2B: Post-intervention speckle tracking echocardiography bull's eye plot at 6 months of follow-up showing improvement in left ventricular global longitudinal strain to −15.4% and left ventricular ejection fraction of 36.6%.

Basic echocardiographic measurements and speckle tracking echocardiography:

Transthoracic echocardiography was performed by an independent observer blinded to the intervention group using EPIQ 7 C (Philips Healthcare, Andover, MA, USA) system. E/e’ ratio was calculated using tissue doppler imaging. LV ejection fraction (LVEF) was determined by modified Simpson's biplane method.^12^ Three standard apical views were obtained in grayscale using a frame rate of 60–100 frames per second of three consecutive cardiac cycles. Offline analysis using the Automated Cardiac Motion Quantification (aCMQ) feature on the Qlab software (QLab Cardiac Analysis ver.10, Philips Healthcare Inc) was done. Mean GLS was calculated by averaging the peak GLS values of the three apical views. A 17-segment polar plot (Bulls' eye) provided visual and quantitative representations of regional LV functions by plotting color-coded values of peak-systolic strain (Fig. 2).^13^ Conventional 2D echocardiography as well as STE were done at baseline, 72 h, and 6 months post intervention.

### 2.2 Statistical analysis

Continuous data was expressed as mean ± standard deviation (SD) and categorical data as proportions. To test for differences in various characteristics, Student's *t*-test was used for continuous variables and the Chi-square test for categorical variables. A *p* value of <0.05 was considered statistically significant. SPSS version 24.0 (IBM Corp, Armonk, NY) software were used for statistical analysis. A written informed consent was taken from each patient. The study protocol was cleared by the Institutional Ethics Committee (IRB number: F.No.17/IEC/MAMC/2018/49).

## 3 Results

During the study period of 18 months, a total of 30 patients with TA and aortic and/or renal involvement were screened, of whom 15 were excluded in view of the poor echocardiographic window.

### 3.1 Baseline characteristics

Table 1 summarizes baseline characteristics of 15 patients (mean age 22.67 ± 5.37 years, 73.3% females). Of the 15 patients, 12 were hypertensive, 3 presented with features of CHF, 6 had reduced ejection fraction (LVEF<50%). Median NT pro BNP was 496 pg/ml (224–1407 pg/ml). Mean ejection fraction, GLS and E/e’ ratio were 43.11 ± 17.42%, −7.18 ± 2.23%, 13.82 ± 2.97 respectively. None of our patients had a raised ESR or CRP prior to intervention or in the follow up period suggesting a quiescent stage of the disease process.

**Table 1:** Baseline demographic, biochemical, and echocardiographic parameters in study subjects (*n* = 15).

Age (mean ± SD) (in years)	22.67 ± 5.37
Female (n) (in %)	11 (73.3)
BMI (mean ± SD) (kg/m^2^)	18.02 ± 1.87
Hypertension (n) (in %)	12 (80)
Median NT pro BNP [IQR](in pg/ml)	496 (224–1407)
Serum creatinine	1.24 ± 0.16 mg/dL
eGFR	72.4 ± 3.4 mL/min
LVEDD (mean ± SD) (in cm)	4.84 ± 0.74
LVESD (mean ± SD) (in cm)	3.48 ± 1.09
LVEDV (mean ± SD) (in ml)	98.27 ± 36.93
LVESV (mean ± SD) (in ml)	60.87 ± 42.42
LVEF(mean ± SD) (in %)	43.11 ± 17.42
E/e’ ratio (mean ± SD)	13.82 ± 2.97
Global longitudinal strain (mean ± SD) (%)	−7.18 ± 2.23
Aortic annulus (mean ± SD) (in cm)	2.48 ± 0.23
Left atrium size (mean ± SD) (in cm)	3.55 ± 0.47
Septal thickness (mean ± SD) (in cm)	1.49 ± 0.19
Posterior wall thickness (mean ± SD) (in cm)	1.62 ± 0.21
Mitral regurgitation (n) (%) Mild Mitral regurgitation (n) (%) Moderate Mitral regurgitation (n) (%) Severe Mitral regurgitation (n) (%)	3 (50.0%) 2 (33.3%) 1 (16.67%) 0
Aortic regurgitation (n) (in %)	0 (0.0%)

Abbreviations: BMI: body mass index; SD: standard deviation; %: percentage; n: number; LVEDD: left ventricle end diastolic dimension; LVEF: left ventricle ejection fraction; LVESD: left ventricle end systolic dimension; LVEDV: left ventricle end diastolic volume; LVESV: left ventricle end systolic volume; eGFR: estimated glomerular filteration rate.

### 3.2 Percutaneous intervention

Percutaneous transluminal balloon angioplasty with stenting (Boston Scientific Express Vascular and Epic Vascular Self Expanding Stent) was done to descending thoracic aorta in three, thoracoabdominal aorta in four, and abdominal aorta in two patients. One patient underwent redo balloon angioplasty of previously stented abdominal aorta. Bilateral renal artery balloon angioplasty was done in two patients and unilateral renal artery balloon angioplasty in three patients. All patients who underwent aortic intervention had residual peak systolic gradient less than 20 mm Hg. After renal balloon angioplasty, none of the patients had residual stenosis more than 20%.

### 3.3 Serial change in blood pressure

Both average systolic and diastolic blood pressure decreased significantly at 72 h post intervention and a further decrease was seen at six months follow up (Supplementary Table 1). Of the 12 hypertensive TA patients, six achieved normal blood pressure (SBP <120 and DBP <80 mm Hg) while other six showed improvement in hypertension.

### 3.4 Serial change in biochemical and echocardiographic parameters

Subgroup analysis was performed by dividing patients intotwo groups based on LVEF (a) reduced LVEF (<50%) and normal LVEF (>50%). Table 2 and Supplementary Table 2 summarize postintervention serial change in biochemical and echocardiographic parameters in these two groups. Heart failure symptoms had improved in all 3 patients. Mean EF increased from 24.62 ± 12.14% to 45.6 ± 9.45% (*p* = 0.001), E/e’ ratio decreased from 15.15 ± 3.19 to 10.8 ± 2.56 (*p* = 0.005). There was a significant improvement in serum creatinine levels pre and post intervention (1.3 ± 0.2 mg/dL vs 0.9 ± 0.6 mg/dL; *p* = 0.04). Similarly, eGFR values had significantly improved post intervention (pre: 72.4 ± 3.4 mL/min vs post: 88.1 ± 2.6 mL/min; *P* < 0.0001). In the normal LVEF group, both GLS and E/e’ were deranged indicating subclinical LV systolic and diastolic dysfunction. NT pro BNP was also elevated though not to the extent seen in the reduced LVEF group. Post-intervention, median NT pro BNP decreased from 512 pg/ml at baseline (80–898.5 pg/ml) to 34 pg/ml (29–70.8 pg/ml) at 6 months (*p* < 0.01), mean GLS improved from −8.80 ± 0.77% to −16.3 ± 0.78% at 6 months (*p* < 0.001) and mean E/e’ decreased from 12.93 ± 2.63 to 7.8 ± 2.73 (*p* = 0.005) at 6 months follow up.

**Table 2:** Post-intervention serial change in biochemical and echocardiographic parameters in reduced ejection fraction group (n=6)

Parameter	Baseline	72 hours post intervention	p value	6 months post intervention	p value
NT pro BNP- median value (IQR) (pg/ml)	1673 (970-2401.5)	1219 (654-1620.8)	**0.01**	80 (40-354)	**0.001**
LVEDD (mean ± SD) (in cm)	5.63 ± 0.40	5.48 ± 0.36	**0.05**	4.80 ± 0.50	**0.005**
LVESD (mean ± SD) (in cm)	4.20 ± 0.75	4.0 ± 0.70	**0.01**	3.12 ± 0.86	**0.008**
LVEDV (mean ± SD) (in cm)	131.83 ± 35.45	119.52 ± 32.30	**0.05**	90.32 ± 30.50	**0.005**
LVESV (mean ± SD) (in cm)	101.17 ± 40.95	87.80 ± 36.90	**0.01**	50.60 ± 31.70	**0.005**
LVEF(mean ± SD) (in %)	24.62 ± 12.14	27.10 ± 11.35	**0.05**	45.6 ± 9.45	**0.001**
E/e’ ratio(mean ± SD)	15.15 ± 3.19	13.50 ± 2.69	**0.01**	10.8 ± 2.56	**0.005**
Global longitudinal strain (mean ± SD) (in %)	−5.49 ± 2.71	−7.20 ± 2.54	**0.01**	-13.1 ± 2.30	**0.005**
Aortic annulus (mean ± SD) (in cm)	2.60 ± 0.32	2.59 ± 0.31	NS	2.43 ± 0.27	NS
Left atrium size (mean ± SD) (in cm)	4.10 ± 0.64	3.99 ± 0.56	NS	3.20 ± 0.55	**0.01**
Septal thickness (mean ± SD) (in cm)	1.47 ± 0.17	1.46 ± 0.18	NS	1.30 ± 0.20	**0.008**
Posterior wall thickness (mean ± SD) (in cm)	1.62 ± 0.23	1.62 ± 0.22	NS	1.35 ± 0.18	**0.005**
Mitral regurgitation (n) (%) Mild Mitral regurgitation (n) (%) Moderate Mitral regurgitation (n) (%) Severe Mitral regurgitation (n) (%)	3 (50.0%) 2 (33.3%) 1 (16.67%) 0	3 (50%) 2 (33.3%) 1 (16.7%) 0	- NS NS NS	1(16.67%) 1 (16.67%) 0 0	- NS NS NS
Aortic regurgitation (n) (%)	0	0	-	0	-

Abbreviations: SD: standard deviation; %: percentage; n: number; LVEDD: left ventricle end diastolic dimension; LVEF: left ventricle ejection fraction; LVESD: left ventricle end systolic dimension; LVEDV: left ventricle end diastolic volume; LVESV: left ventricle end systolic volume

## 4 Discussion

Left ventricular dysfunction in TA is multifactorial with both inflammation and hemodynamic factors being implicated. Findings of our pilot study revealed significant improvement in LV function determined using conventional and speckle tracking echocardiography following aortic/renal interventions in TA. Observational studies have reported relatively high prevalence of LV dysfunction in TA of 15–50%.^14^, ^15^, ^16^ In our study, 6 out of 15 patients (40%) had LV systolic dysfunction (LVEF <50%) at baseline of whom three had CHF. In a previous study by Tyagi et al,^9^ 25.7% of children with renovascular hypertension undergoing intervention presented with severe LV systolic dysfunction. Post-intervention improvement in hypertension, LV systolic function, and heart failure symptoms have been reported in few case reports and series.^6^^,^^7^^,^^17^ In the present study, patients with low LVEF showed marked improvement in LV dimensions and LV volumes. Mean ejection fraction increased from 24.62 ± 12.14% to 45.6 ± 9.45% (*p* = 0.001) at six months follow up. However, in one patient, there was no improvement in LV ejection fraction and the LV cavity remained dilated at six months despite disease remission. This could be related to the onset of irreversible LV dysfunction due to diffuse and prolonged disease process. This underscores the concept of early detection of LV dysfunction and timely intervention before the irreversible damage.

Conventional echocardiography generally detects LV dysfunction following prolonged disease process when dysfunction is overt while GLS obtained by STE is a sensitive and validated method for detection of subclinical LV dysfunction.^18^ At baseline, LV systolic dysfunction was present in six patients only while none had normal GLS value indicating subclinical LV dysfunction in the rest of the patients. GLS is governed by the subendocardial myofibers which are the earliest and most commonly affected in conditions associated with increased after load like coarctation of aorta and aortic stenosisdue to compromised subendocardial blood flow reserve secondary to LV hypertrophy.^19^, ^20^, ^21^ Furthermore, prolonged systole due to increased afterload is translated into shorter diastole thereby further compromising the diastolic myocardial perfusion time. The combination of the above factors over a period of time increase myocardial stiffness leading to a deranged GLS in the earlier stage which over a period of time culminates into overt LV dysfunction. These findings are supported by pre and post-interventional data on GLS in patients with aortic stenosis. In a study done by Poulsen et al,^22^ GLS was significantly reduced in patients with aortic stenosis and preserved LVEF and it improved following aortic valve replacement (baseline −9 ± 4%vs −14 ± 4% at 12 months, *p* < 0.001). To the best of our knowledge, there is no published data regarding the role of GLS in patients with TA patients. GLS has the potential to identify Takayasu arteritis patients who might benefit from early intervention in order to preserve the LV function.

Diastolic dysfunction was present in all patients irrespective of the systolic function. E/e’ ratio decreased significantly at 72 h (*p* < 0.01) and a further decrease was seen at 6 months (*p* = 0.005) indicating significant improvement in diastolic function. On subgroup analysis,the reduced LVEF group though showed significant improvement in mean E/e’ ratio, though it didn't reach normal value (<8). This suggests that there may be a subset of patients in whom pressure load has lead to myocardial changes, including hypertrophy and myocardial fibrosis, as well as alterations in active relaxation that are less reversible. To the best of our knowledge, there is no literature available on post-intervention change in diastolic function in Takayasu arteritis patients. These findings could be extrapolated from data onpatients with congenital AS.^23^

The potential limitations of the our study was a small sample size with shorter duration of follow up. However, this was a pilot study in a small group of patients demonstrating the feasibility of STE in TA. This calls for future studies in larger patient sub-groups in order to validate these findings. Additionally, cardiac MRI or endomyocardial biopsy was not carried out to rule out myocarditis in these patients.

## 5 Conclusion

Overt LV dysfunction and congestive heart failure are known entities in Takayasu arteritis. Hemodynamic factors by increasing the afterload have important role in LV dysfunction. Timely correction of these hemodynamic factors by appropriate endovascular/surgical intervention can lead to symptomatic as well as objective improvement in myocardial contractile performance/myocardial systolic function indices. 2D STE is a useful modality to assess subclinical systolic dysfunction in these patients. Early intervention can improve hypertension, ejection fraction, diastolic function and prevent subclinical LV dysfunction to convert into overt dysfunction.

## Funding

None.

## Declaration of competing interest

Authors have no conflict of interest to disclose.
