# Supplementary material for: Left ventricular function assessment after aortic and renal intervention in Takayasu arteritis by speckle tracking echocardiography: A pilot study
Source: Indian Heart J. 2022 Feb 23;74(2):139–43. doi: 10.1016/j.ihj.2022.02.004 (PMC9039681; doi:10.1016/j.ihj.2022.02.004)
Supplement: Multimedia component 1 [file mmc1.docx]

**Supplementary Table 1: Post-intervention serial change in average systolic and diastolic blood pressure in study subjects (n=15)**

|  | **Baseline** | **72 hours post intervention** | **P value** | **6 months post intervention** | **P value** |
| --- | --- | --- | --- | --- | --- |
| Average Systolic Blood Pressure (mm Hg) (mean ± SD) | 178.6±22 | 142.2±14 | **0.01** | 128.4±11 | **0.001** |
| Average Diastolic Blood Pressure (mm Hg) (mean ± SD) | 108.3±12 | 90.9±9 | **0.01** | 77.9±8 | **0.001** |

**Abbreviations:** SD: standard deviation
