# Supplementary material for: Left ventricular function assessment after aortic and renal intervention in Takayasu arteritis by speckle tracking echocardiography: A pilot study
Source: Indian Heart J. 2022 Feb 23;74(2):139–43. doi: 10.1016/j.ihj.2022.02.004 (PMC9039681; doi:10.1016/j.ihj.2022.02.004)
Supplement: Multimedia component 2 [file mmc2.docx]

**Supplementary Table 2: Post-intervention serial change in biochemical and echocardiographic parameters in normal ejection fraction group (n=9)**

| **Parameter** | **Baseline** | **72 hours post procedure** | **p value** | **6 months post procedure** | **p value** |
| --- | --- | --- | --- | --- | --- |
| Median NT pro BNP [IQR] (pg/ml) | 512 (80-898.5) | 380 (68- 594.7) | NS | 34 (29-70.8) | **0.01** |
| LVEDD (mean ± SD) (in cm) | 4.31±0.25 | 4.30±0.23 | NS | 4.2±0.27 | NS |
| LVESD (mean ± SD) (in cm) | 2.73±0.41 | 2.72±0.40 | NS | 2.66±0.52 | NS |
| LVEDV (mean ± SD) (in cm) | 75.89±13.88 | 74.40±12.60 | NS | 73.8±10.60 | NS |
| LVESV (mean ± SD) (in cm) | 34.0±8.47 | 33.6±7.8 | NS | 33.2±8.10 | NS |
| Ejection Fraction (mean ± SD) (%) | 55.44±3.38 | 55.40±3.40 | NS | 56.80±4.12 | NS |
| E/e’ ratio (mean ± SD) | 12.93±2.63 | 11.18±2.50 | **0.01** | 7.8±2.73 | **0.005** |
| Global longitudinal strain (mean ± SD) (%) | -8.80±0.77 | -11.10±0.69 | **0.01** | -16.3±0.78 | **<0.001** |
| Aortic annulus (mean ± SD) (in cm) | 2.49±0.14 | 2.48±0.12 | NS | 2.45±0.17 | NS |
| Left atrium size (mean ± SD) (in cm) | 3.2±0.10 | 3.16±0.16 | NS | 2.75±0.14 | **0.01** |
| Septal thickness (mean ± SD) (in cm) | 1.51±0.22 | 1.50±0.20 | NS | 1.18±0.25 | **0.004** |
| Posterior wall thickness (mean ± SD) (in cm) | 1.62±0.22 | 1.60±0.20 | NS | 1.22±0.28 | **0.004** |
| Mitral regurgitation (n) | 0 | 0 | - | 0 | - |
| Aortic regurgitation (n) | 0 | 0 | - | 0 | - |

**Abbreviations:** SD: standard deviation; %: percentage; n: number; LVEDD: left ventricle end diastolic dimension; LVEF: left ventricle ejection fraction; LVESD: left ventricle end systolic dimension; LVEDV: left ventricle end diastolic volume; LVESV: left ventricle end systolic volume
